# Odyssey of an International Medical Trainee

**DOI:** 10.1007/s11606-025-10011-z

**Published:** 2025-11-12

**Authors:** Divyansh Agarwal

**Affiliations:** Department of Surgery, Massachusetts General Hospital, Boston, MA USA

“I wish our hospital had a better support system for international physician trainees,” I expressed to the attending surgeon near the half-way mark of a long cytoreductive surgery. Disconcerted by recent changes in immigration policies, I felt that convenient access to an immigration liaison for advice could put much of the unrest among international trainees at ease. Whether it was the length of the surgery or reflection of a larger leadership crisis, the seriousness of the attending surgeon’s response took me by surprise: “Well, you all can get deported anytime,” insinuating that such initiatives might be wasted efforts. A lengthy silence followed.

In the months that followed, the mere fact of being an international student provoked guilt. Despite making up 23–25% of the US physician workforce today, being a vulnerable, visa-requiring surgeon was not welcome in that operating theater. I struggled to reconcile that conversation with the purported core values of academic excellence and mentorship that many hospitals, including ours, pride themselves on. That one moment in the operating room, evocative of the microaggressions that immigrant trainees often face, was enough to dent years of self-belief and confidence.

As I grapple with the rapidly changing dynamics affecting practicing physicians in North America, I draw comfort from a more recent encounter with a different surgeon in the same operating theater.

Scrubbing in a thyroidectomy for a young woman, a professional opera singer, the attending surgeon asked me about my postgraduation plans. Excited to learn that I was interviewing for fellowship positions, our conversation assumed the form of a mock interview. Over the next 30 min, we took meticulous care to protect the nerves that would allow her to perform *Habanera* from Carmen in a month. I marveled at the tortuous path of the recurrent laryngeal nerve as it hugged the parathyroid glands, and the thyroid gland was dissected off the trachea.

“What’s the hardest thing you’ve done in residency?”, our mock interview progressed. I thought for a moment and recounted a fascinating operative case I led few months ago. Almost as if he expected a different response, my attending asked “You have not been home in over six years, right? What could be harder than being away from family?”

As we approximated the muscles supporting the hyoid bone, we bonded over our aging parents and family abroad. Before leaving the operating room, the attending remarked how surgical exposure made the case relatively easy. “Remember as you progress through your career—the most important thing for a surgeon is exposure”.

In that moment, those words took on a deeper meaning. Indeed, operative success follows once there is good exposure in the surgical field. Yet, exposure to mentors who might share a background with you, share your culture or interests, or have navigated similar challenges of the US immigration system is just as critical to set trainees up for success in the long term. While exposure to challenging operative cases or clinical patient encounters helps you grow as a physician, when geopolitical changes “expose” international trainees, it leaves them vulnerable. As international trainees on visas, this tension is our lived reality. Faced with recent uncertainty on our legal status as visa holders, I've begun to think of exposure not only in terms of valuable training experiences and mentorship but also in terms of vulnerability.

Training as a surgeon in the USA often reminds me of The Odyssey. The Greek king of Ithaca survived a shipwreck, and spurred on by tragedy navigated his path home. With recent uncertainty in the USA with respect to changes in work visas and immigration, physicians with international roots find ourselves caught between Scylla and Charybdis. One seemingly benign act of advocacy, intellectual expression, or of merely leaving the USA to visit family could spiral into a monstrous whirlpool like Charybdis. On the other hand, providing care to those in need while living through microaggressions and the constant fear of whether our homes or lives might be uprooted after training is no less than battling a six-headed sea monster. International trainees strive to steer clear of both monsters while keeping excellence and patient duties at the center of our decisions. Our journey mimics Odysseus’ wherein the path requires navigating a series of tests and risking loss in service to a larger goal.

On this journey, it is imperative that our host programs maintain their support and commitment, now more than ever, for vulnerable immigrant trainees. Too few people wonder what healthcare would look like if visa-holding healthcare providers and staff simply vanished one day: healthcare would likely collapse, and the invisible fence of protection around certain respected professions would disappear. I cannot begin to imagine how physician shortages would expound at my own hospital system and its grave implications for patient care. Future international trainees with visa requirements should not hesitate from discussing immigration issues openly. Are H1B, J1, O1, TN1, and EB1 just an alphabet soup to your program’s leadership? Do they understand the visa process intricacies and provide meaningful support? In my program, international trainees were made aware of pro bono legal services offered by the city of Boston. While no program is perfect, having an appreciation for these details helps trainees make a more informed decision about which program they may want to call home during a period when they may not get to see their family.

In medicine and in life, the most important thing is exposure to people who carry the moral courage to support others when it matters the most. As another residency application cycle draws near, trainees deserve to be in an environment where they can find their people—where suggesting an open dialogue of support for international trainees is not chastised. Two entirely different operative experiences as a senior surgical resident taught me the impact we can have in nurturing younger talents. Leaders who will leave a lasting impact, like the culture hero of *Odyssey*, will not shy away from sailing through challenging terrains, and leading the next generation to a better horizon.

